# Bridging the gap: opportunities for transitions of care pharmacist review of outpatient parenteral antimicrobial therapy prescriptions prior to hospital discharge

**DOI:** 10.1017/ash.2024.52

**Published:** 2024-04-18

**Authors:** Sara Stashluk, Michelle Ramos, Tyla Carettini, James B. Cutrell, Seana Mathew, Marguerite Monogue, Jennifer Nguyen, James M. Sanders, Esther Y. Golnabi

**Affiliations:** 1 Department of Pharmacy, University of Texas Southwestern Medical Center, Dallas, Tx, USA; 2 Department of Medicine, Division of Infectious Diseases and Geographic Medicine, University of Texas Southwestern Medical Center, Dallas, Tx, USA

## Abstract

**Purpose:**

Pharmacist-led initiatives providing optimization of medications during transitions of care (TOC) have shown to have a positive impact on prescribing practices and patient outcomes. This study aims to evaluate the role and impact of TOC pharmacist review of outpatient parenteral antimicrobial therapy (OPAT) prescriptions prior to hospital discharge.

**Methods:**

In a retrospective chart review, patients with OPAT prescriptions between November 1, 2022 and January 31, 2023 were evaluated using prescription-specific and intervention-specific data points. Prescription-specific data points included intravenous antimicrobials prescribed, indication, prescribing team, and time from OPAT prescription to TOC pharmacist review. Intervention-specific data points included antimicrobial optimization (dose/frequency, duration, and other), prescription clarification, and laboratory monitoring.

**Results:**

Of the 137 OPAT prescriptions evaluated, 67 required intervention by TOC pharmacists (48.9%). The General Infectious Disease Consult team placed 71.5% of OPAT prescriptions and required interventions less frequently (42.9%) compared to the other teams. Antimicrobial optimization interventions accounted for 54.2% of interventions, which were primarily related to medication dose and frequency.

**Conclusion:**

The TOC pharmacists can play a key role in the evaluation of OPAT prescriptions at hospital discharge. This intervention demonstrated how TOC pharmacists can effectively collaborate with the OPAT team, which builds on prior evidence of the role and value of pharmacists in the transitional care setting.

## Background

Outpatient parenteral antimicrobial therapy (OPAT) is a treatment strategy that allows patients to receive intravenous (IV) antimicrobials in the outpatient setting for the management of an infectious disease (ID).^1^ This strategy has shown to reduce length of hospital stay and costs and improve patient quality of life.^2^ Past publications have shown that transitions of care (TOC) pharmacists play a key role in reviewing discharge IV and oral medication prescriptions at discharge, including increasing optimal antimicrobial prescribing and decreasing hospital readmission.^3,4^ This study aims to evaluate the role and impact of TOC pharmacist review specific to OPAT prescriptions prior to hospital discharge.

## Methods

### Setting

The University of Texas Southwestern Medical Center (UTSW) is an academic medical center consisting of two university hospitals with 825 beds. The OPAT program was established in August 2020 to provide dedicated management of patients receiving IV antimicrobial therapies in the outpatient setting. The OPAT program consists of an ID-trained pharmacist, a nurse coordinator, and an ID physician. Due to limited resources, the OPAT program currently only manages patients who are internally referred by ID providers, which represents ∼70% of the total OPAT patients discharged from the hospital per an internally conducted data collection study. In 2023, the TOC pharmacists reviewed 105 OPAT prescriptions per month on average.

The UTSW TOC pharmacist team consists of 15 TOC pharmacists to provide various services, including pre-discharge bedside education, discharge medication review, and discharge medication counseling. Thirteen of the 15 TOC pharmacists are located in the hospitals with three dedicated to patients who recently underwent solid organ transplantation and two who are dedicated to patients discharging from the emergency department (ED). This hospital team provides coverage every day (weekdays: 7 a.m.–7:30 p.m.; weekends: 7 a.m.–5:30 p.m.; ED: 2 p.m.–12 a.m.). The TOC pharmacists have been integrated into the OPAT workflow since early 2021. The TOC OPAT prescription queue is constantly reviewed by at least one TOC pharmacist during the TOC hours. The expectation is for the OPAT prescriptions to be reviewed by a TOC pharmacist within 30 minutes of prescription entry. Timing of OPAT prescription verification and documenting of the verification as a chart note are dependent on intervention(s) needed and the timeliness of the provider response.

**Table 1.** Characteristics and frequencies of outpatient parenteral antimicrobial therapy (OPAT) prescription and transitions of care (TOC) pharmacist interventions

Characteristics of OPAT prescriptions	
**OPAT indications, n (%)**	Total = 189
Bloodstream infection	53 (28.0)
Bone and joint infection	32 (16.9)
Intra-abdominal infection	26 (13.8)
Pulmonary infection	19 (10.1)
Urinary tract infection	17 (9.0)
Endovascular infection	16 (8.5)
Skin and soft tissue infections	12 (6.3)
Other	14 (7.4)
**Prescribed intravenous antimicrobials, n (%)**	Total = 172
Daptomycin	32 (18.6)
Ceftriaxone	29 (16.9)
Cefazolin	17 (9.9)
Vancomycin	17 (9.9)
Piperacillin-tazobactam	15 (8.7)
Cefepime	14 (8.1)
Ertapenem	13 (7.6)
Other^a^	35 (20.3)
**Prescribing teams, n (%)**	Total = 137
General infectious diseases consult	98 (71.5)
Transplant infectious diseases consult	7 (5.1)
Cystic fibrosis	2 (1.5)
Other^b^	30 (21.9)
**TOC pharmacist interventions**	
**Total number of prescriptions reviewed, n**	137
**Prescriptions requiring intervention, n (%)**	67 (48.9)
**Prescribing inpatient teams (expressed as prescriptions needing intervention/total prescriptions (%))**	
General infectious diseases consult	42/98 (42.9)
Transplant infectious diseases consult	4/7 (57.1)
Cystic fibrosis	2/2 (100)
Other (no infectious diseases or cystic fibrosis consult)	19/30 (63.3)
**Type of interventions**	Total = 72
** Antimicrobial optimization, n (%)**	**39 (54.2)**
Dose/frequency-related	21 (29.2)
Duration-related	6 (8.3)
Other^c^	12 (16.7)
** Prescription clarification, n (%)**	**16 (22.2)**
** Laboratory monitoring, n (%)**	**14 (19.4)**
Addition/deletion of vancomycin monitoring labs	6 (8.3)
Addition of creatinine phosphokinase (CPK) for patients on daptomycin	2 (2.8)
Change from basic metabolic panel (BMP) to comprehensive metabolic panel (CMP)	1 (1.4)
Other	5 (6.9)
** Other, n (%)**	**3 (4.2)**
**Average time from OPAT prescription entry to completion of TOC pharmacist’s note**	198 min
Average with exclusion of four extreme outliers	62 min

a Meropenem (8), ampicillin-sulbactam (5), penicillin G (3), ganciclovir (3), micafungin (2), fluconazole (2), ceftazidime-avibactam (2), cefoxitin (2), amikacin (2), metronidazole (2), levofloxacin (1), ceftazidime (1), ceftaroline (1), ampicillin (1)

b Solid organ transplant (21), pulmonary (2), internal medicine (2), digestive and liver disease (2), bone marrow transplant (1), colorectal surgery (1), and geriatrics (1)

c Reasons for other antimicrobial-optimization interventions included discharge antimicrobial(s) mismatching with hospital-administered antimicrobial(s) (7), available alternative antimicrobial(s) with better ease of administration (2), duplicate spectrum of activity in antimicrobials prescribed (1), drug–drug interaction (1), and missing supportive hydration (1)

The inpatient providers prescribe IV medications for discharge by utilizing a Care Coordination order set. Within the order set, providers are expected to include the following details for each prescription: medication name, dose, route, frequency, intended duration of therapy, end date, and laboratory monitoring parameters. After a provider submits a prescription for outpatient IV antimicrobial(s), the order is routed to the TOC OPAT prescription queue. A TOC pharmacist then ensures that the IV antimicrobial(s) is/are prescribed in accordance with institution-specific OPAT recommendations, including guidance for medication dosing and laboratory monitoring. If edits are needed, the TOC pharmacists intervene and work with providers within the treatment team to update the prescription. After the prescription deemed to be appropriate, the TOC pharmacists verifiy the prescription and acknowledge their review in a note documented in the electronic medical record (Epic^®^). This note instructs the inpatient care coordinators to start the discharge medication set-up process.

Education specific to OPAT was provided to the TOC pharmacist team by an ID-trained OPAT pharmacist on a quarterly basis for a year following the initial TOC integration. Since then, the education is given on an as-needed basis. Communication about any changes to the institution-specific OPAT guidance is regularly communicated to the TOC pharmacist team, and the OPAT team is available throughout the day (weekdays 8 a.m.–5 p.m.) for questions regarding OPAT prescriptions. Once the OPAT prescription is verified by a TOC pharmacist, the OPAT team continues to monitor the patient on a daily basis (with the exception of weekends) until discharge and assumes full management of IV antimicrobial therapies after the patient is discharged from the hospital.

### Design

This retrospective chart review included patients with Care Coordination orders containing IV antimicrobial prescriptions at UTSW that were entered between November 1, 2022, and January 31, 2023. Patient information was collected through the iVent feature on Epic, where pharmacists can categorize and document interventions made regarding patient care. The patient was excluded if the treatment plan did not include an IV antimicrobial, if the antimicrobial therapy was completed during inpatient admission, or if the admission resulted in a self-directed discharge. From the initial list of patients meeting the eligibility criteria, the data collector reviewed every second patient. Prescription-specific and intervention-specific data points were collected using REDCap (Research Electronic Data Capture).^5^ The prescription-specific data points included admission and discharge dates, date and time of OPAT prescription, date and time of TOC OPAT review, prescribing provider team (General ID, Transplant ID, Cystic Fibrosis, and other teams), indication for OPAT, and type(s) of intervention(s) made by TOC pharmacists. The TOC pharmacists’ interventions were categorized as following: safety monitoring laboratory parameters, prescription clarification, antimicrobial optimization, and other for interventions outside of these categories. Antimicrobial optimization interventions were further classified into dose/frequency-related, duration-related, drug allergy clarification, or other interventions. The UTSW Human Research Protection Program determined that this quality improvement study did not require Institutional Review Board oversight.

## Results

A total of 282 prescriptions with iVents were identified between November 1, 2022 and January 31, 2023. Every second patient was then assessed for eligibility, which yielded a total of 137 OPAT prescriptions. The most frequent OPAT indications were bloodstream infections (28.0%), bone and joint infections (16.9%), and intra-abdominal infections (13.8%). The total number of OPAT indications exceeded the total number of OPAT prescriptions as 41 patients had more than one indication for OPAT (eg, a bloodstream infection secondary to a urinary tract infection). The most frequent OPAT antibiotics used were daptomycin (18.6%), ceftriaxone (16.9%), cefazolin (9.9%), and vancomycin (9.9%). Of the 137 prescriptions, 67 prescriptions (48.9%) required intervention by the TOC pharmacists. The General ID team prescribed the highest number of OPAT prescriptions (71.5%) and had the lowest rate of prescriptions requiring intervention (42.9%). Of the 67 interventions, approximately half were regarding the antimicrobial dose, with the most frequent intervention being related to dose/frequency of antimicrobials (29.2%) followed by duration of therapy (8.3%). The other interventions (4.2%) included clarification of an OPAT prescription the patient was not previously receiving inpatient, recommendation for alternative antimicrobial to improve ease of administration, avoidance of duplication of therapy, and avoidance of drug–drug interactions. Prescription clarification interventions (22.2%) were primarily consisted of typographical errors and missing information. Missing information included any information necessary to execute a complete and valid prescription, including dose, route, and frequency. Of the 14 laboratory interventions, six (8.3%) involved addition or deletion of vancomycin monitoring labs, two (2.8%) involved addition of creatinine phosphokinase for patients on daptomycin, and one (1.4%) involved changing laboratory monitoring from basic metabolic panel (BMP) to complete metabolic panel (CMP). The average time from Care Coordination order submission to TOC pharmacist verification was 198 minutes. Excluding the four outliers, the average time from prescription submission to TOC review was 62 minutes. These outliers occurred due to providers’ making incorrect selections in the Care Coordination order, which resulted in delay in TOC pharmacists receiving notification of the new OPAT prescriptions.

## Discussion

The study demonstrates that the integration of TOC pharmacist review into the OPAT prescribing process aided in optimizing medication regimens in approximately half of all OPAT prescriptions placed during the study period. Although this study did not examine patient outcomes past discharge, it is anticipated that the errors, if not acted upon by TOC pharmacists, may have resulted in patient harm, potentially even readmissions. According to a systematic review by Morabet *et al*, the rate of hospital readmissions due to medication-related problems ranges from 3% to 64%, with approximately 64% of these problems having been preventable.^6^ Furthermore, long-term IV antimicrobial therapies are associated with increased risk of complications with ∼18% related to antimicrobials themselves.^7^

Despite prescribing most OPAT prescriptions, the General ID team required the least number of TOC pharmacist interventions. This is likely due to the use of the standardized ID documentation process of prescribing OPAT using a template (Supplementary Material) that is separate from the Care Coordination order set. The inclusion of ID pharmacists during the multidisciplinary General ID rounds on weekdays and a concomitant review of the prescriptions by the OPAT program members also potentially contributed to the lower percentage of TOC pharmacist interventions.

The inpatient care coordinators at UTSW require a pharmacist review of the OPAT prescriptions to start discharge set-up process. Given the high volume of discharging OPAT patients at the institution and limited dedicated OPAT personnel, the integration of the TOC team into the workflow of reviewing the OPAT prescriptions was necessary to provide timely review of prescriptions. Many OPAT programs report lack of personnel resources.^8^ The study findings demonstrate that the integration of TOC pharmacist into the OPAT prescription review may be an attractive option for other OPAT programs to consider implementing. Despite an average of 62 minutes from OPAT prescription to verification of prescription via pharmacist’s chart note, most time was spent awaiting responses from providers to correct errors and/or ensure prescription was complete. Therefore, the addition of OPAT verification does not largely add to the TOC pharmacist’s workload or delay patient discharge.

Limitations of this study include a relatively small sample size and the lack of comparison between pre- and post-implementation of the TOC pharmacist OPAT review process. Post-discharge rates of adverse drug events, ED visits, or hospital readmissions were not measured, which could substantiate the impact of the TOC interventions.

TOC pharmacists play a vital role in managing and reviewing drug therapy prior to hospital discharge. Expanding their role to include OPAT prescription review improves timeliness of prescription reviewed while ensuring prescription accuracy and completeness. Although the incorporation of TOC pharmacists into the OPAT prescribing process is not well described in literature, this study illustrates the opportunity and feasibility of establishing such a process.

## Supporting information

Stashluk et al. supplementary material Stashluk et al. supplementary material

## Supplementary material

For supplementary material accompanying this paper visit http://doi.org/10.1017/ash.2024.52. click here to view supplementary material

## Research transparency and reproducibility

None to provide.
