# Supplementary material for: Bridging the gap: opportunities for transitions of care pharmacist review of outpatient parenteral antimicrobial therapy prescriptions prior to hospital discharge
Source: Antimicrob Steward Healthc Epidemiol. 2024 Apr 18;4(1):e50. doi: 10.1017/ash.2024.52 (PMC11036442; doi:10.1017/ash.2024.52)
Supplement: Stashluk et al. supplementary material [file S2732494X24000524sup001.docx]

**Supplementary Material**

**Templated Phrase Used by General Infectious Diseases Team to Order Outpatient Parenteral Antimicrobial Therapies**

Home IV Abx Orders:

Rx: ***

Weekly *** faxed to ###-###-####

Anticipated end date: ***

Please ensure the following imaging is ordered: *** no later than ***

Please arrange followup with UTSW ID clinic no later than: ***

PICC line care

Make sure **all questions** from Infusion Pharmacy, Nursing Agency directed to Infectious Diseases Clinic, ###-###-####

Ordering MD: ***

UTSW ID clinician assuming responsibility: Dr. XXX

IF the patient remains in-house by the date of recommended ID followup, please ensure that the ID consult service is contacted to re-evaluate the patient.
